# Supplementary material for: Single-gene resolution of diversity-driven overyielding in plant genotype mixtures
Source: Nat Commun. 2023 Jun 8;14:3379. doi: 10.1038/s41467-023-39130-z (PMC10250416; doi:10.1038/s41467-023-39130-z)
Supplement: Supplementary file 1 — Supplementary Information [file 41467_2023_39130_MOESM1_ESM.pdf]

Supplementary Information

Single-gene resolution of diversity-driven overyielding in plant genotype mixtures

Samuel E. Wuest*[1,2,3,4], Lukas Schulz[5], Surbhi Rana[6], Julia Frommelt[1], Merten Ehmig[7], Nuno D. Pires[2], Ueli Grossniklaus[2], Christian S. Hardtke[6], Ulrich Hammes[5], Bernhard Schmid[1,3] and Pascal A. Niklaus[1]

Supplementary Figures

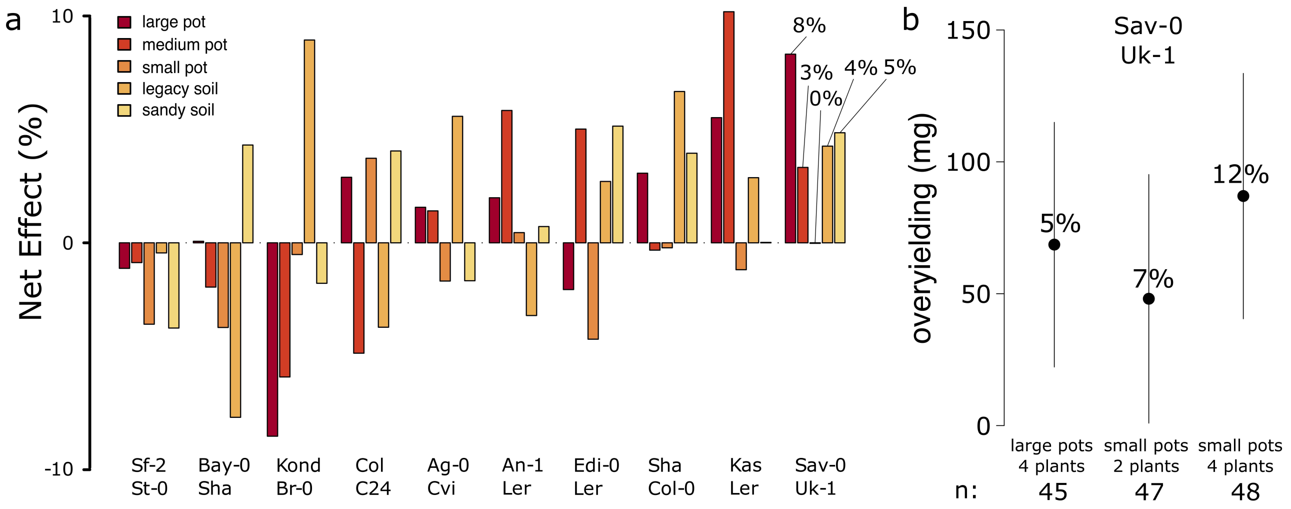

Supplementary Figure 1 A screen for consistent genotypic diversity effects between divergent *Arabidopsis* accession pairs. a Shown are estimates of net overyielding (observed mixture yield compared with average yields of component monocultures) of ten *Arabidopsis* accession pairs across different soil types or pot sizes. For each estimate, seven pots (large pot, medium pot, small pot) or five pots (legacy soil, sandy soil) of each monoculture and the mixture were sown, resulting in a total of 930 pots containing four plants each. Note that both consistent negative (left) or consistent positive (right) effects appear. Furthermore, a soil-by-diversity interaction in the Bay-0 * Sha combination has been examined in more detail previously[1]. **b** Confirmation of consistently positive genotypic diversity effects in the genotype combination Slavice-0 (Sav-0) and Umkirch-1 (Uk-1) under three different conditions. Shown are estimated net overyielding for each condition, number above points indicate the relative net effect (%). Error bars: +/- s.e.m. Source data are provided as a Source Data file.

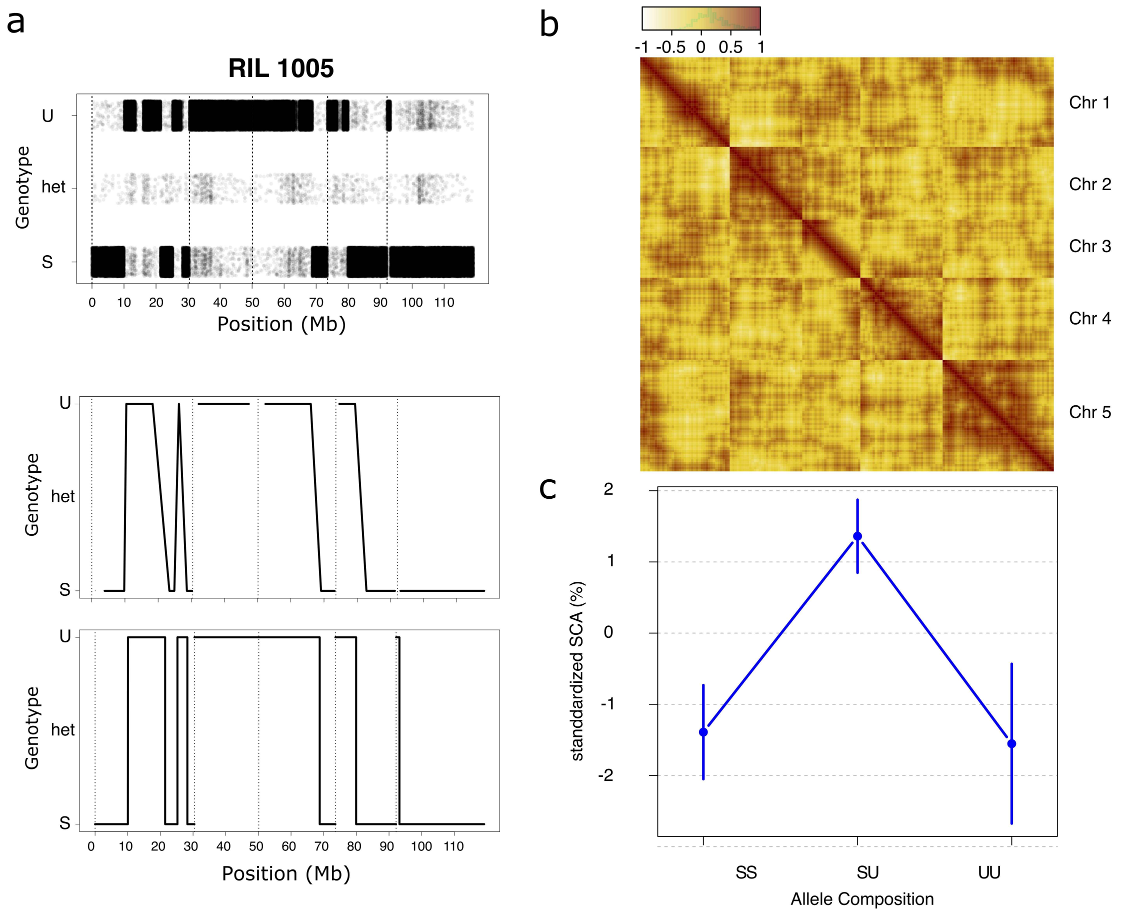

Supplementary Figure 2. Reconstruction of RIL genotypes from low-coverage genome re-sequencing and QTL effect sizes. a Top: Genotype calls across the genome in RIL US1005; and comparison of molecular markers (middle) and genotype reconstruction based on low-coverage genome re-sequencing (Viterbi-Path, bottom). **b** Correlations of allelic compositions between all markers and across all genotype combinations **c** Effect of allelic composition on specific combining abilities at the QTL chromosome 2 (QTL2, bottom). SS n = 66; UU n = 28; SU n = 96. Error bars: +/- s.e.m. Source data are provided as a Source Data file.

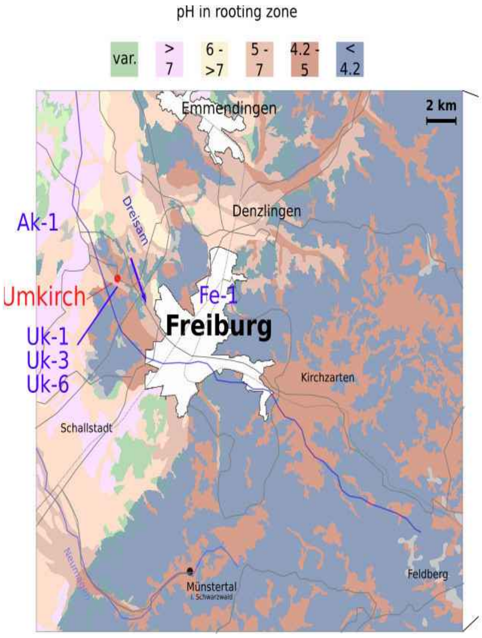

Supplementary Figure 3. Soil acidity map of the southern black forest region, the area in which the Uk-1 accession was collected. Transect sampling performed by Shindo and colleagues[2]: purple arrow. Data from http://maps.lgrb-bw.de/. var = variable

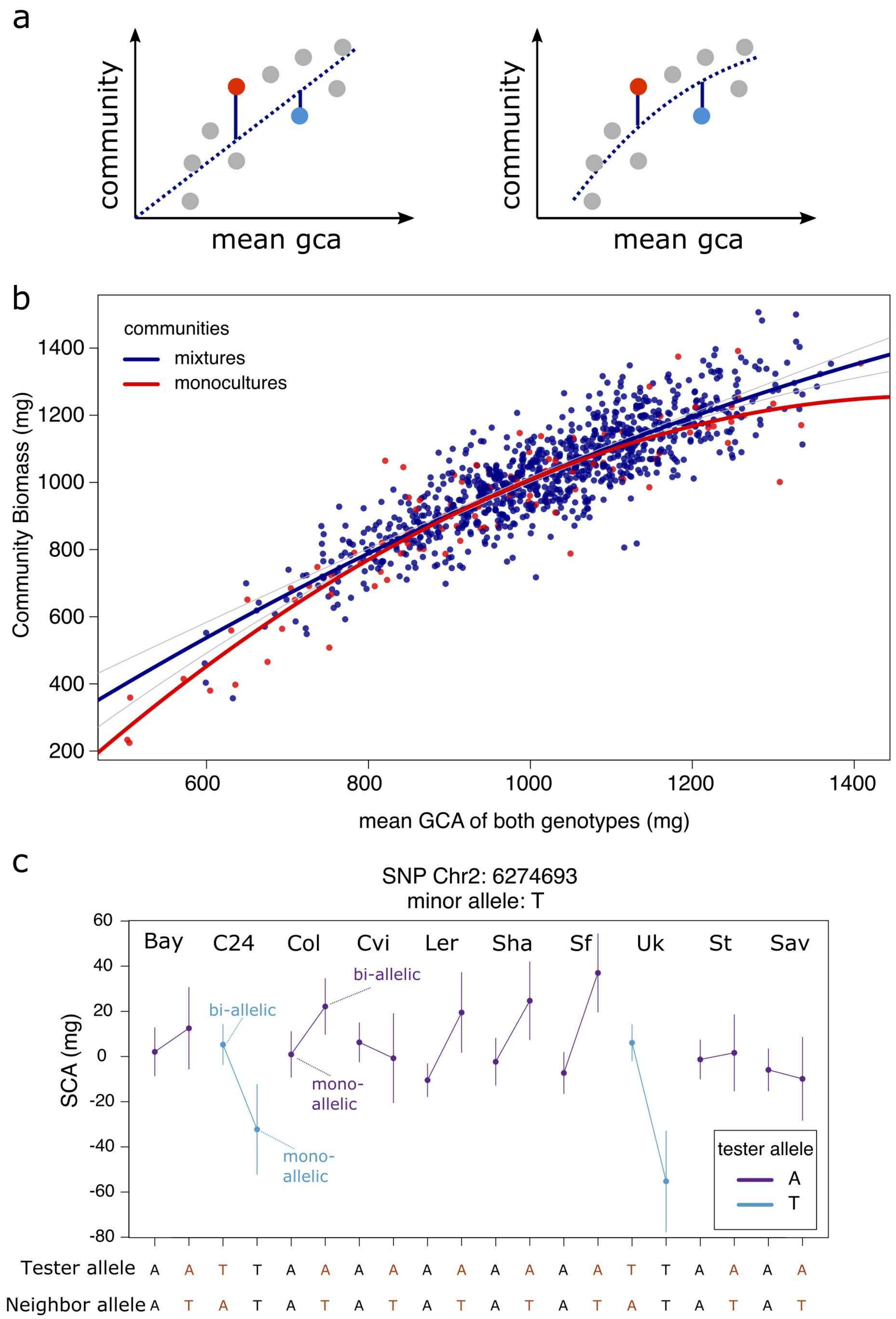

Supplementary Figure 4: Determination of SCAs in factorial (tester-associate) competition design for GWAS and SCA across different tester lines and the different allelic diversity levels at a SNP within *AtSUC8*. a Specific combining ability of a genotypic composition is typically estimated from deviates of observed community productivities from expectations (in this case, the average GCA of both genotypes); however, because different compositions varied so strongly in total productivities, the relationship between the mean GCA of a genotype composition and the

overall community productivity might become non-linear (e.g., driven by increasingly restricted space for combinations of highly productive genotypes). In this case, such a systematic relationship can first be modeled, and the SCA estimated as the deviation from this modeled relationship. **b** Observed relationship between the average GCA of a genotype composition and its community productivity. **c** Uk-1 and C24 both carry the minor (T) allele at SNP Chr2-6274693. When combined with genotypes also carrying the minor allele, the resulting mixtures show on average lower SCA, when combined with genotypes carrying the major allele (A), they exhibit on average higher SCA. Dots denote means (AA-combinations n = 77; TT-combinations n = 20 all testers except for Uk-1 and C24, where AA-combinations n = 78 and TT-combinations = 19). Error bars: +/- s.e.m. Source data are publicly available through the Zenodo data respository (DOI: 10.5281/zenodo.6983283).

Supplementary Table 1: Descriptions of protein-coding genes found within the QTL on chromosome 2.

Locus	Description	Symbols
AT2G14378	Encodes a ECA1 gametogenesis related family protein	NA
AT2G14390	Hypothetical protein	NA
AT2G14440	Leucine-rich repeat protein kinase family protein	NA
AT2G14460	Hypothetical protein	NA
AT2G14500	F-box family protein	ATFDB14
AT2G14510	Leucine-rich repeat protein kinase family protein	NA
AT2G14520	CBS domain protein (DUF21)	NA
AT2G14530	Encodes a member of the TRICHOME BIREFRINGENCE-LIKE gene family	TBL13
AT2G14540	Serpin 2	SRP2; ATSRP2
AT2G14560	Encodes LURP1, a member of the LURP cluster (late upregulated in response to Hyaloperonospora parasitica). LURP1 is required for full basal defense to H. parasitica.	NA
AT2G14580	Pathogenesis related protein, encodes a basic PR1-like protein.	PRB1; ATCAPE7; ATPRB1
AT2G14610	PR1 gene expression is induced in response to a variety of pathogens. It is a useful molecular marker for the SAR response. Expression of this gene is salicylic-acid responsive.	PR1; ATCAPE9
AT2G14620	Xyloglucan endotransglucosylase/hydrolase 10	XTH10
AT2G14635	ARABIDILLO protein	NA

AT2G14660	Thymocyte nuclear-like protein	NA
AT2G14670	Sucrose-proton symporter 8	SUC8; AtSUC8

Supplementary Table 2: Conditions and experiment sizes of Sav-0/Uk-1 mixture experiments (screen/confirmation experiments)

**Conditions** (soil type, pot size, no of plants/pot)	**Monoculture pots**	**Mixture pots**	**Overyielding (%)**	**glht-contrast p-value**
ED73, **large** pots, 4 plants/pot	7	7	8%	0.0645
ED73, **medium** pots, 4 plants/pot	7	7	3%	0.5263
ED73, **small** pots, 4 plants/pot	7	7	0%	0.9963
**sand-rich** soil, medium pots, 4 plants/pot	5	5	5%	0.3922
**legacy soil**, medium pots, 4 plants/pot	5	5	4%	0.5003
ED73, **large** pots, **4** plants/pot	12	24	5%	0.1421
ED73, **small** pots, **2** plants/pot	12	24	7%	0.3101
ED73, **small** pots, **4** plants/pot	12	24	12%	0.0641

Supplementary References

1. Wuest, S. E. & Niklaus, P. A. A plant biodiversity effect resolved to a single chromosomal region. *Nat. Ecol. Evol.* **2**, 1933–1939 (2018).
2. Shindo, C., Bernasconi, G. & Hardtke, C. S. Intraspecific competition reveals conditional fitness effects of single gene polymorphism at the Arabidopsis root growth regulator BRX. *New Phytol.* **180**, 71–80 (2008).
