# Supplementary material for: Single-gene resolution of diversity-driven overyielding in plant genotype mixtures
Source: Nat Commun. 2023 Jun 8;14:3379. doi: 10.1038/s41467-023-39130-z (PMC10250416; doi:10.1038/s41467-023-39130-z)
Supplement: Supplementary file 3 — Reporting Summary [file 41467_2023_39130_MOESM3_ESM.pdf]

nature portfolio

Corresponding author(s): Samuel Wuest

Last updated by author(s): May 11, 2023

Reporting Summary

Nature Portfolio wishes to improve the reproducibility of the work that we publish. This form provides structure for consistency and transparency in reporting. For further information on Nature Portfolio policies, see our Editorial Policies and the Editorial Policy Checklist.

Statistics

For all statistical analyses, confirm that the following items are present in the figure legend, table legend, main text, or Methods section.

n/a	Confirmed	
☐	☒	The exact sample size (*n*) for each experimental group/condition, given as a discrete number and unit of measurement
☐	☒	A statement on whether measurements were taken from distinct samples or whether the same sample was measured repeatedly
☐	☒	The statistical test(s) used AND whether they are one- or two-sided<br>*Only common tests should be described solely by name; describe more complex techniques in the Methods section.*
☐	☒	A description of all covariates tested
☐	☒	A description of any assumptions or corrections, such as tests of normality and adjustment for multiple comparisons
☐	☒	A full description of the statistical parameters including central tendency (e.g. means) or other basic estimates (e.g. regression coefficient) AND variation (e.g. standard deviation) or associated estimates of uncertainty (e.g. confidence intervals)
☐	☒	For null hypothesis testing, the test statistic (e.g. *F*, *t*, *r*) with confidence intervals, effect sizes, degrees of freedom and *P* value noted<br>*Give P values as exact values whenever suitable.*
☒	☐	For Bayesian analysis, information on the choice of priors and Markov chain Monte Carlo settings
☒	☐	For hierarchical and complex designs, identification of the appropriate level for tests and full reporting of outcomes
☐	☒	Estimates of effect sizes (e.g. Cohen's *d*, Pearson's *r*), indicating how they were calculated

Our web collection on statistics for biologists contains articles on many of the points above.

Software and code

Policy information about availability of computer code

Data collection	no software was used for data collection
Data analysis	R version 4.2.0; bwa version 0.7.16a; samtools v1.5; MPR package (in R) v0.1; Fiji/ImageJ v 2.0.0-rc-65/1.52a; SnapGene version 6.1; multcomp package version 1.4-19 (in R)

For manuscripts utilizing custom algorithms or software that are central to the research but not yet described in published literature, software must be made available to editors and reviewers. We strongly encourage code deposition in a community repository (e.g. GitHub). See the Nature Portfolio guidelines for submitting code & software for further information.

Data

Policy information about availability of data

All manuscripts must include a data availability statement. This statement should provide the following information, where applicable:

- Accession codes, unique identifiers, or web links for publicly available datasets
- A description of any restrictions on data availability
- For clinical datasets or third party data, please ensure that the statement adheres to our policy

Original data: The datasets described are available through the Zenodo data repository [https://doi.org/10.5281/zenodo.7896146]; Data re-use: The raw data of the association study have been published on the Zenodo data repository [https://doi.org/10.5281/zenodo.6983283]. Sequencing data were deposited in the NCBI Sequence Read Archive, BioProject PRJNA967174 [https://www.ncbi.nlm.nih.gov/bioproject/PRJNA967174].

Research involving human participants, their data, or biological material

Policy information about studies with human participants or human data. See also policy information about sex, gender (identity/presentation), and sexual orientation and race, ethnicity and racism.

Reporting on sex and gender	N/A
Reporting on race, ethnicity, or other socially relevant groupings	N/A
Population characteristics	N/A
Recruitment	N/A
Ethics oversight	N/A

Note that full information on the approval of the study protocol must also be provided in the manuscript.

Field-specific reporting

Please select the one below that is the best fit for your research. If you are not sure, read the appropriate sections before making your selection.

☐ Life sciences ☐ Behavioural & social sciences ☒ Ecological, evolutionary & environmental sciences

For a reference copy of the document with all sections, see nature.com/documents/nr-reporting-summary-flat.pdf

Ecological, evolutionary & environmental sciences study design

All studies must disclose on these points even when the disclosure is negative.

Study description	The study describes the genetic mapping of allelic diversity effects on mixture overyielding in experimental plant populations of Arabidopsis thaliana. Overyielding was first determined in a set of 10 genotype pairs by comparing genotype mixture biomass productivity (above-ground dry matter mass) across five different conditions (5-7 replicates per composition and soil-type or pot size, resulting in 930 measurements). After the genotype pair Slavice-0 (Sav-0) and Umkirch-1 (Uk-1) had consistently exhibited positive overyielding estimates in this screen and in a follow-up experiment across three different densities, a population of 18 recombinant inbred lines (RILs) and the two parents were grown in all pairwise combinations (half-diallel design, 210 unique genotype compositions) to genetically map positive effects of allelic diversity across the genome on specific combining ability (SCA, a measure of community overyielding). This experiment was replicated across four blocks, and the genomes of the RILs were reconstructed through shallow whole-genome resequencing. This experiment revealed one major-effect quantitative trait locus (QTL) of allelic diversity on overyielding on chromosome two. In an orthogonal mapping analysis, data from a previous study were re-used, where ten Arabidopsis thaliana genotypes (including Sav-0 and Uk-1) had been grown in competition with each of a set of 98 natural accessions, in a full-factoral manner and replicated across two complete blocks (980 genotype compositions + all monocultures). An association of positive allelic single nucleotide polymorphism diversity on SCA was detected within the identified QTL - specifically, at the AtSUC8 gene, encoding a root-expressed proton-sucrose-symporter. The gene (and the sequence polymorphisms between Sav-0 and Uk-1) was then characterized using molecular and biochemical methods (molecular: Sanger sequencing of genomic fragment amplified by PCR; biochemical: sucrose transport assays in oocytes, comparing the two protein variants, n = 9-10; see Materials and Methods), and effects of polymorphisms on root growth under different substrate pH-conditions measured (plate assays, pH 6.8 and 4.8, six seeds per genotype and pH). The latter experiment was performed using 80 Sav-0 x Uk-1 RILs, individually genotyped for allelic identity at the AtSUC8 locus.
Research sample	Natural inbred accessions of Arabidopsis thaliana (Slavice-0 and Umkirch-1 publicly available through stock centers, e.g. www.arabidopsis.info, and 96 additional accessions for the association mapping and initial screen), and a sample of 80 recombinant inbred lines derived from a cross between the Sav-0 and Uk-1. From these RILs, 18 were used for QTL mapping, the rest were used for root growth assays on plates.
Sampling strategy	Sample size calculations were not performed for the QTL mapping, since the genetic architecture of the identified diversity effect was not known previous to the experiment. For the association mapping based on re-used data, the sampling and experimental design was determined in the original publication.
Data collection	Molecular/biochemical data were collected as described in detail in the Methods section. Above-ground plant biomass was harvested destructively and dried at 65°C for at least three days before weighing. Data collection of plant experiments was performed primarily by Samuel E Wuest, with some help by Merten Ehmig, Nicole Ponta and Daniela Stöckli (plant handling and some weighing). The phenotypic data were typed into an Excel-sheet. For root length measurements, scanned plate images were loaded into Fiji, where primary roots were manually traced using the freehand tool and then measured using the "measure"-function.
Timing and spatial scale	For plant experiments, pots were grown in a greenhouse, and each pot (either 5.5x5.5x6 cm, 6x6x5.5 cm, 7x7x8 cm or 9x9x10 cm) consisted of a community (2-4 plants, either one or two genotypes per composition). Seeds were sown directly on soil (multiple seeds per position within a pot) and between 6-10 days after sowing continuously thinned until only one healthy seedling remained

	per position. Plants were grown until approx. 5% the earliest flowering genotypes in a given block exhibited approx. 5–10 dehiscent siliques on the main flowering stem (between 42 and 51 days after sowing).
Data exclusions	No data were excluded, except when individual plants within a given experimental plant stand died (then, the whole pot was excluded from the analyses)
Reproducibility	All pot experiments can be considered orthogonal reproductions of the same positive diversity effect. Supplementary Figures 1 a and b show independent experiments with positive overyielding estimates for Sav-0 / Uk-1 mixtures (7 out of 8 estimates positive, though individual estimates are not statistically different from zero). Figure 2 shows that a positive diversity effect between genotypes containing Sav-0 or Uk-1 alleles occurs at the level of a QTL on chromosome 2, in an independent set of genotypes (RILs derived from a Sav-0 x Uk-1 cross). Figure 3 shows results from another, independent association experiment where allelic differences at a single SNP within this QTL drives significant overyielding, tested across a set of 980 different mixtures. Oocyte assays and root growth assays were not replicated independently.
Randomization	Experimental units (pots) within blocks or experiments were completely randomized after sowing. Throughout experiments, pots were re-positioned randomly within trays or tables every 7–10 days.
Blinding	Blinding was ensured by the use pot number codes as the primary sample identifier and non-interpretable genotype line codes. In general, in genetic mapping experiments using natural accessions or Recombinant Inbred Lines (RILs), genotype composition does not reveal information about the specific allelic composition at a specific marker or genomic location, essentially excluding the possibility of experimenter biases to influence the measurements.

Did the study involve field work? ☐ Yes ☒ No

Reporting for specific materials, systems and methods

We require information from authors about some types of materials, experimental systems and methods used in many studies. Here, indicate whether each material, system or method listed is relevant to your study. If you are not sure if a list item applies to your research, read the appropriate section before selecting a response.

Materials & experimental systems

n/a	Involved in the study
☒	☐ Antibodies
☒	☐ Eukaryotic cell lines
☒	☐ Palaeontology and archaeology
☒	☐ Animals and other organisms
☒	☐ Clinical data
☒	☐ Dual use research of concern
☐	☒ Plants

Methods

n/a	Involved in the study
☒	☐ ChIP-seq
☒	☐ Flow cytometry
☒	☐ MRI-based neuroimaging

Dual use research of concern

Policy information about dual use research of concern

Hazards

Could the accidental, deliberate or reckless misuse of agents or technologies generated in the work, or the application of information presented in the manuscript, pose a threat to:

No	Yes
☒	☐ Public health
☒	☐ National security
☒	☐ Crops and/or livestock
☒	☐ Ecosystems
☒	☐ Any other significant area

Experiments of concern

Does the work involve any of these experiments of concern:

No	Yes	
☒	☐	Demonstrate how to render a vaccine ineffective
☒	☐	Confer resistance to therapeutically useful antibiotics or antiviral agents
☒	☐	Enhance the virulence of a pathogen or render a nonpathogen virulent
☒	☐	Increase transmissibility of a pathogen
☒	☐	Alter the host range of a pathogen
☒	☐	Enable evasion of diagnostic/detection modalities
☒	☐	Enable the weaponization of a biological agent or toxin
☒	☐	Any other potentially harmful combination of experiments and agents
